# A fingertip-type magnetic pulse detection device for unusual monitoring conditions

**DOI:** 10.1038/s44172-023-00102-2

**Published:** 2023-07-28

**Authors:** Haoxuan Shen, Xiaoyi Gu, Yihong Wu

**Affiliations:** 1 Department of Electrical and Computer Engineering, National University of Singapore, Singapore, 117583 Singapore; 2 NUS (Chongqing) Research Institute, Chongqing Liang Jiang New Area, Chongqing, 401123 China

**Keywords:** Biomedical engineering, Electrical and electronic engineering

## Abstract

Wearable devices for continuous non-invasive monitoring of physiological signals are crucial for preventive care and management of chronic conditions. However, these devices are either sensitive to skin conditions or require external stimuli, such as light or electrical excitation, to penetrate the skin for pulse detection. This often results in large motion artifacts and unsuitability for certain skin conditions. Here we demonstrate a simple fingertip-type device which can detect clear pulse signals under a range of conditions, including fingers covered by opaque substances such as a plaster or nail polish, or fingers immersed in water. The structure of the device consists of a pair of magnets and a magnetic sensor. Both experimental and numerical results show that the detected pulsation signals correspond directly to the vibrations induced by blood circulation with negligible influence from modulated magnetic signature of blood. Therefore, this device could be used in conditions which are challenging for existing pulse detectors and might also have its application extended to blood pressure measurement.

## Abstract (web summary)

Shen and colleagues report a fingertip-type magnetic device for pulse detection and show that it works in conditions which might interfere with more conventional pulse detection stimuli, typically light or electric fields. The direct correspondence of the device signals to blood circulation induced vibrations hint also at potential utility in blood pressure measurements.

## Introduction

Continuous non-invasive monitoring of physiological signals such as heart rate, blood pressure and blood oxygen level are crucial for preventive care and management of chronic conditions^1,2^. A plethora of devices have been developed for this purpose, notably photoplethysmography (PPG) that can detect the blood volume changes in the microvascular bed of tissue and arteries through measuring the intensity variation of either transmitted or reflected light from the tissue^3^. From the detected optical signals, we can extract physiological signals such as heart rate and blood oxygen level^4^. Due to its high cost-performance and ease of use, the PPG has become the de facto standard non-invasive vital sign monitoring device in both clinical and non-clinical settings. Despite the high penetration rate, the PPG has several drawbacks such as motion artifacts, skin tone effect, and unsuitability for circumstances where the skin is covered by opaque materials (e.g., plaster or nail polish). Therefore, there is always a need to develop non-optical detectors which can replace or supplement the PPG.

One of the promising techniques that can potentially overcome some of the difficulties facing by the PPG is the magnetoplethysmography (MPG)^5,6^. The early work on MPG was motivated by the fact that oxygenated hemoglobin (HbO_2_) and de-oxygenated hemoglobin (Hb) exhibit different magnetic properties; the former is diamagnetic whereas the latter is of paramagnetic nature^7^. Phua et al. have demonstrated a wrist-type MPG device consisting of a permanent magnet and a Hall sensor which can clearly detect the pulsatile signals in both in vitro and in vivo configurations^5,6^. The authors attributed the MPG signals to modulated magnetic signature of blood (MMSB)^6,8^, based on the hypothesis that the magnetic field of permanent magnet affects the blood flow dynamics, which in turn perturbs the magnetic field distribution in the surrounding area. Since then several research groups have demonstrated MPG prototype devices using either the giant magnetoresistance or Hall sensor which successfully detected the pulsatile signals, demonstrating good reproducibility of the detection method^9–16^. Considering the difference in magnetic responses of HbO_2_ and Hb, the initially proposed MMSB mechanism seems plausible. However, given the small difference (on the order of several ppm) in magnetic susceptibility between HbO_2_ and Hb^7^, it remains controversial whether the MPG signal originates entirely or in part from the MMSB mechanism. Simulations by Sinatra revealed that the disturbance to magnetic field caused by the magnetic properties of blood is on the order of 10^−5^, much smaller than the earth’s magnetic field^17^, although it shows close correlation with the blood flow velocity. The in-vitro experiments performed by Zhang et al. suggest that the MPG signal is mainly derived from the mechanical disturbance of the sensor detection axis caused by blood flood, but quantitative discussion is lacking^18^. Similar results were also reported by Li et al., though it is not clear if the results were obtained from in-vivo or in-vitro measurements^19^. Without a proper understanding of the detection mechanism, it would be difficult to further develop the MPG for practical applications.

Here, we propose a fingertip-type magnetic device which allows to measure the magnetic and vibrational signals simultaneously using a magnetic sensor and a laser Doppler vibrometer (note: the vibrometer is only used to facilitate mechanism studies, it is not part of the magnetic sensing system). Unlike all previous experiments which place a single magnet on the radial artery, we place two magnets on the opposite sides of a fingertip where the mutual attraction between the magnets improves the stability of both magnetic and vibrational signals. We found that the magnetic and vibrational signals are closely correlated with each other, and the average deviation between the two signals is less than 5%. Since the magnetic signal is diminishingly small when the magnets are not in contact with the skin, we can safely conclude that the detected signal is dominated by vibration of the magnet, and the contribution from the MMSB mechanism is negligible. Both analytical and numerical simulation results are in good agreement with the experimental observations. We show that such kind of device can detect clear pulsation signals under all finger conditions—a task that has proven to be rather challenging for existing pulse detectors. Since the measured signal is not originated from the MMSB mechanism, hereafter we call it magnetically detected vibration sensor (MDVS) instead of MPG.

## Results and discussion

**Fig. 1:**
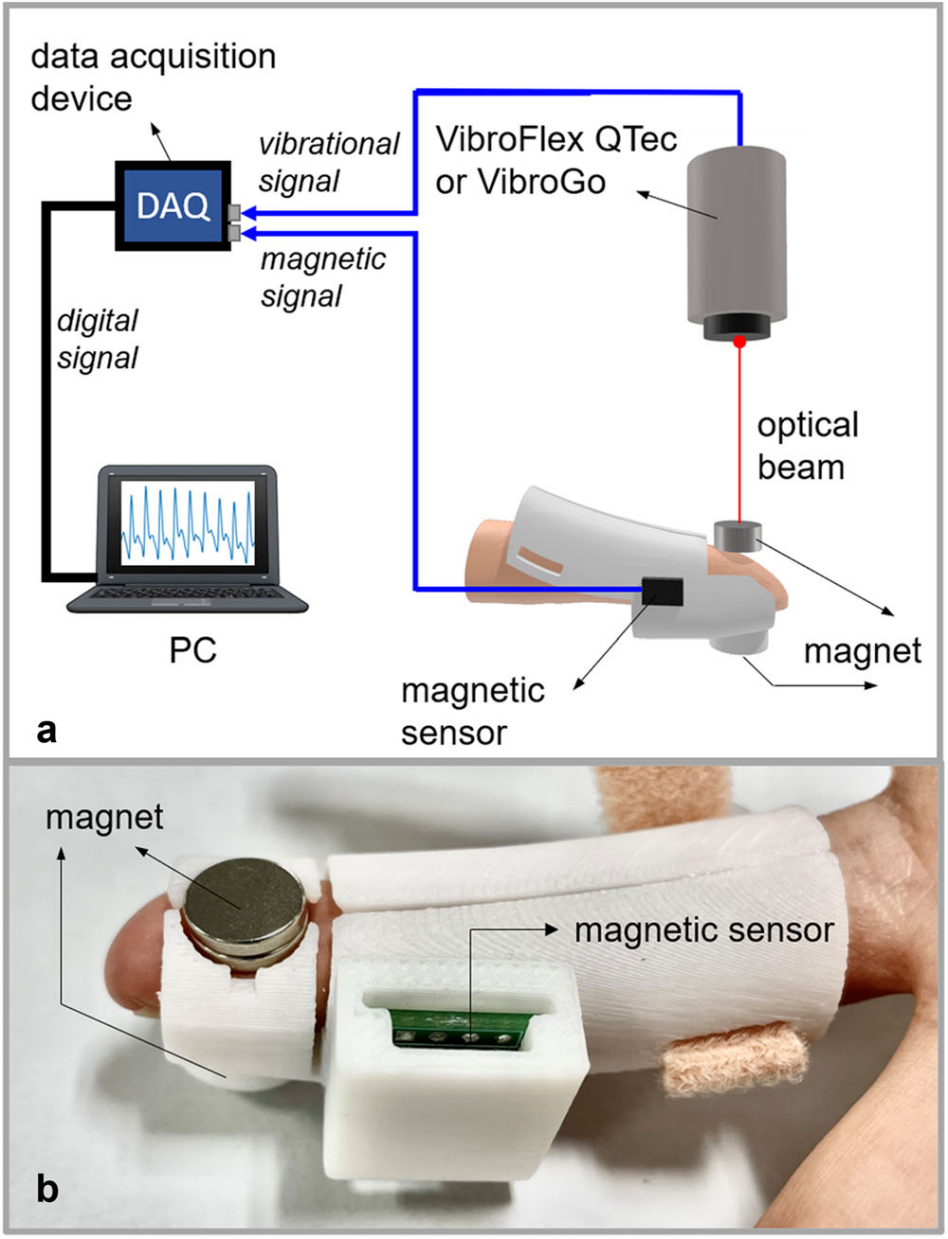
Schematic of the fingertip-type MDVS device and measurement setup. **a** Measurement setup. **b** Photograph of assembled fingertip-type MDVS.

The MDVS device used in this work consists of two cylindrical magnets attached to a fingertip, one of which is firmly attached to a mechanical fixture and the other is movable in response to blood flow-induced skin movement (Fig. 1). A magnetic sensor is placed near the movable magnet (approximately 1.5 cm), which detects changes in the magnetic field of the magnet. Depending on the magnet chosen and positioning of the sensor, various types of magnetic sensors can be used, including the recently developed spin-orbit torque enabled magnetic sensor (Supplementary Fig. 1 in Supplementary Note 1)^20–22^. To facilitate mounting of the sensor, the data presented in this paper are collected using a commercial tunnel magnetoresistance sensor with a dynamic range around 100 Oe and sensitivity of 1.28 mV/Oe (TMR2005 from MultiDimension Technology). The magnets used are N35 NdFeB permanent magnets with a saturation magnetization ${M}_{s}$ = 962.9 kA/m and a coercivity around 12 kOe. The diameter and thickness of the magnets are 10 and 5 mm, respectively. The position and sensing direction of the sensor are determined by the actual field distribution surrounding the magnets (more details are given in the simulation section). The prototype is designed such that the movable magnet can be directly probed by a laser Doppler meter (Polytec VibroGo). The VibroGo signal acquisition unit has two input channels which can be used to measure the vibration and magnetic signals simultaneously. This greatly facilitates the comparison of two signals as the time difference caused by measurement electronics is presumably negligible. The experiments were performed on multiple fingers of healthy subjects of different gender and age. Good reproducibility has been obtained in all measurements conducted.

### Synchronized detection of magnetic and vibration signals and statistical analysis

**Fig. 2:**
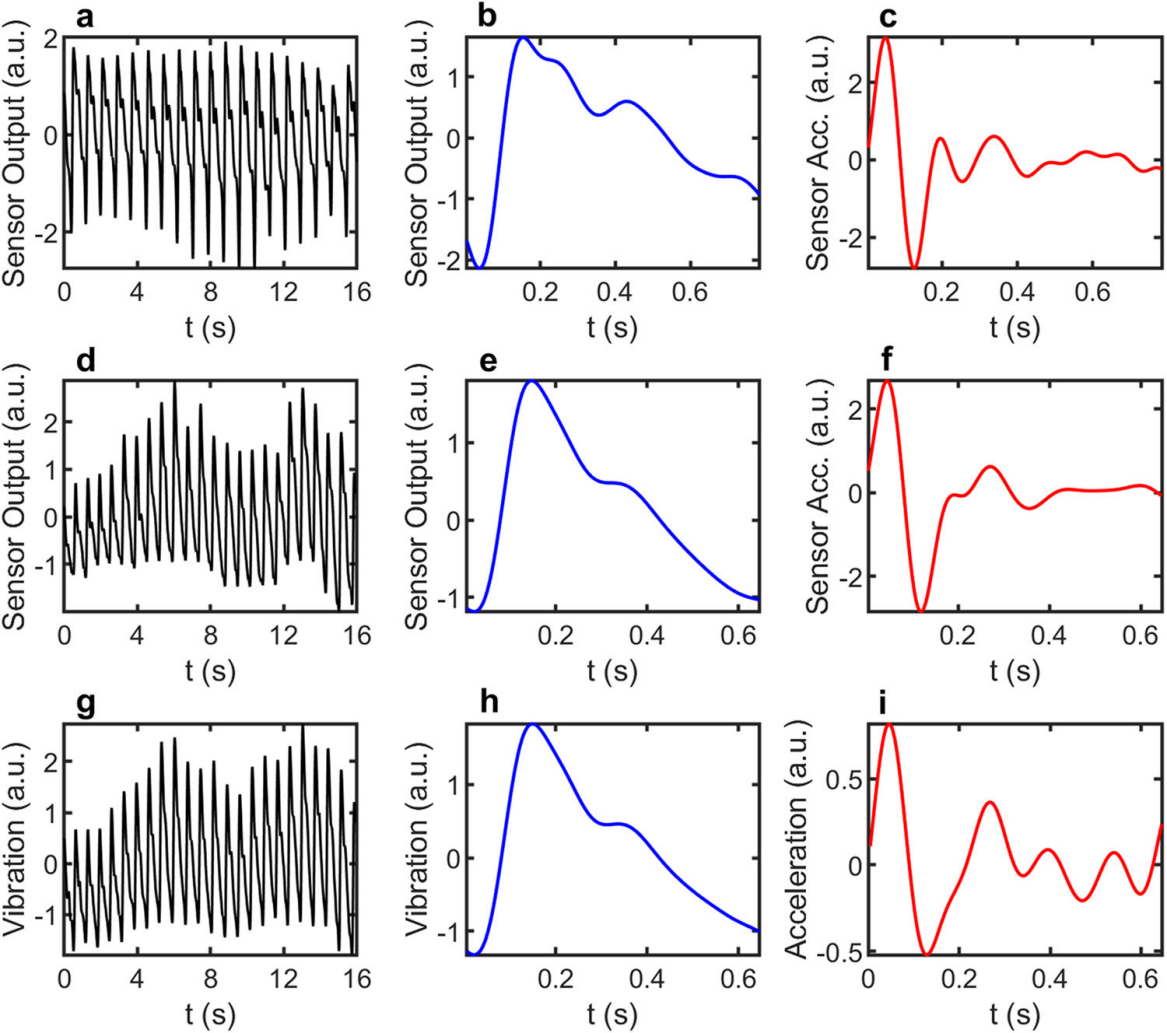
Magnetic and vibrational signals obtained from two healthy subjects. **a**–**c** are signals obtained from subject A: **a** magnetic sensor output signal for a duration of 16 s, **b** average single pulse signal from (**a**), and **c** second derivative of (**b**). **d**–**f** are the corresponding magnetic signals measured from subject B. For comparison, **g**–**i** shows the vibrational signals measured simultaneously with **d**–**f**: **g** is the time-dependent displacement signal, **h** is the average single pulse signal and **i** is the average acceleration detected directly by the vibrometer.

Figure 2 shows the typical magnetic and vibrational signals obtained simultaneously from two healthy subjects (See Supplementary Fig. 2 for more data from different subjects). Figure 2a is the pulsatile signal of subject A (male at late 50s) measured by a magnetic sensor for a duration of 16 seconds. The aspiratory baseline shift has been removed and the data have been smoothed with a bandpass filter with a bandwidth of 0.8–10 Hz. Comparison with commercial PPG sensor confirms that the periodicity of the signal corresponds to the heartbeat rate of the subject. Figure 2b shows the corresponding average single pulse signal with the mean subtracted out and normalized by the standard deviation. The overall shape resembles well the PPG waveform with clear systolic peak, dicrotic notch, and diastolic peak^3^. A high degree of similarity with PPG is also seen in the acceleration pulse waveform (or second derivative), as shown in Fig. 2c, which is useful for evaluating cardiovascular conditions^23^. Figure 2d–f show the same set of data for subject B (female at early 20s). The basic characteristics are the same as those obtained from Subject A, though we do see some subtle differences in the shape of the pulse and acceleration waveform. These results demonstrate clearly that the magnetic signals are reproducible and of good quality. We now turn to the vibrational signals acquired simultaneously by VibroGo from subject B. The pulsatile signals showing in Fig. 2g, h are the time-dependent displacement signals, whereas Fig. 2i is the acceleration signal measured directly by the Doppler meter. Apparently, there is a close correlation between the magnetic and vibrational signals in terms of both periodicity and waveform including the characteristic peaks. The subtle difference in systolic peak position is presumably due to the different time response of the two measurement channels. The correlation coefficient is in the range of 0.9527–0.9924, which is even higher for the averaged single pulse.

### Pulse monitoring under multiple conditions

**Fig. 3:**
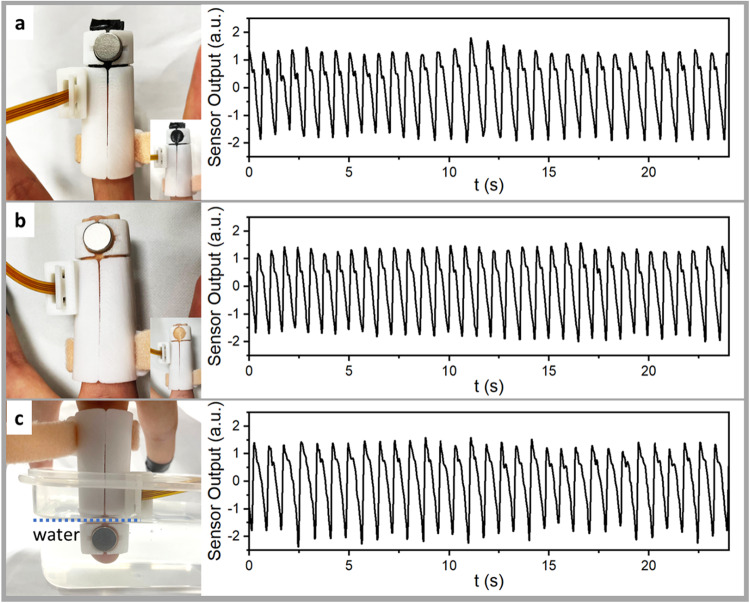
Pulse signals measured under diffident conditions. **a** Fingertip wrapped by a black tape, **b** fingertip wrapped by a plaster, and **c** fingertip immersed in water. Left panel: photography of the MDVS and fingertip during measurement; right panel: measured signals.

To show the pulse monitoring capability of the device under unusual conditions, we have conducted the measurements for fingers under different conditions: (a) wrapped by a black tape, (b) wrapped by a plaster, and (c) immersed in water, and the results are shown in Fig. 3a–c, respectively. As shown in the left panel of Fig. 3a, b, the contact area of the skin and magnet is completely covered by a black tape or a plaster. The former completely blocks the light whereas the latter is semi-transparent. Should a PPG sensor be used for the detection, one would not expect any measurable signal in (a) or a clear signal in (b). But, as shown in the right panel of Fig. 3a, b, clear signals are obtained in both cases, indicating that the black tape or plaster has very little effect to the MDVS detector. To demonstrate that the MDVS is immune to body fluid too, we conducted the measurement by immersing the fingertip and magnets in water, while leaving the magnetic sensor in air (since it is not made waterproof). As shown in the right panel of Fig. 3c, again we can obtain very clear pulse signals. Although the test was done using clear water, we expect that similar results can be obtained when there is body fluid such as sweat and blood on the finger. Repeated experiments show that the signals detected under all the three conditions are stable and reproducible, with clear systolic peak, dicrotic notch, diastolic peak, and equal peak-to-peak span. There is very little difference compared to the results obtained from bare hand in air. These results clearly demonstrate the advantages of MDVS over existing pulse detectors such as PPG, in coping with challenging circumstances.

### Bland–Altman plot analysis of magnetic and vibration signal

**Fig. 4:**
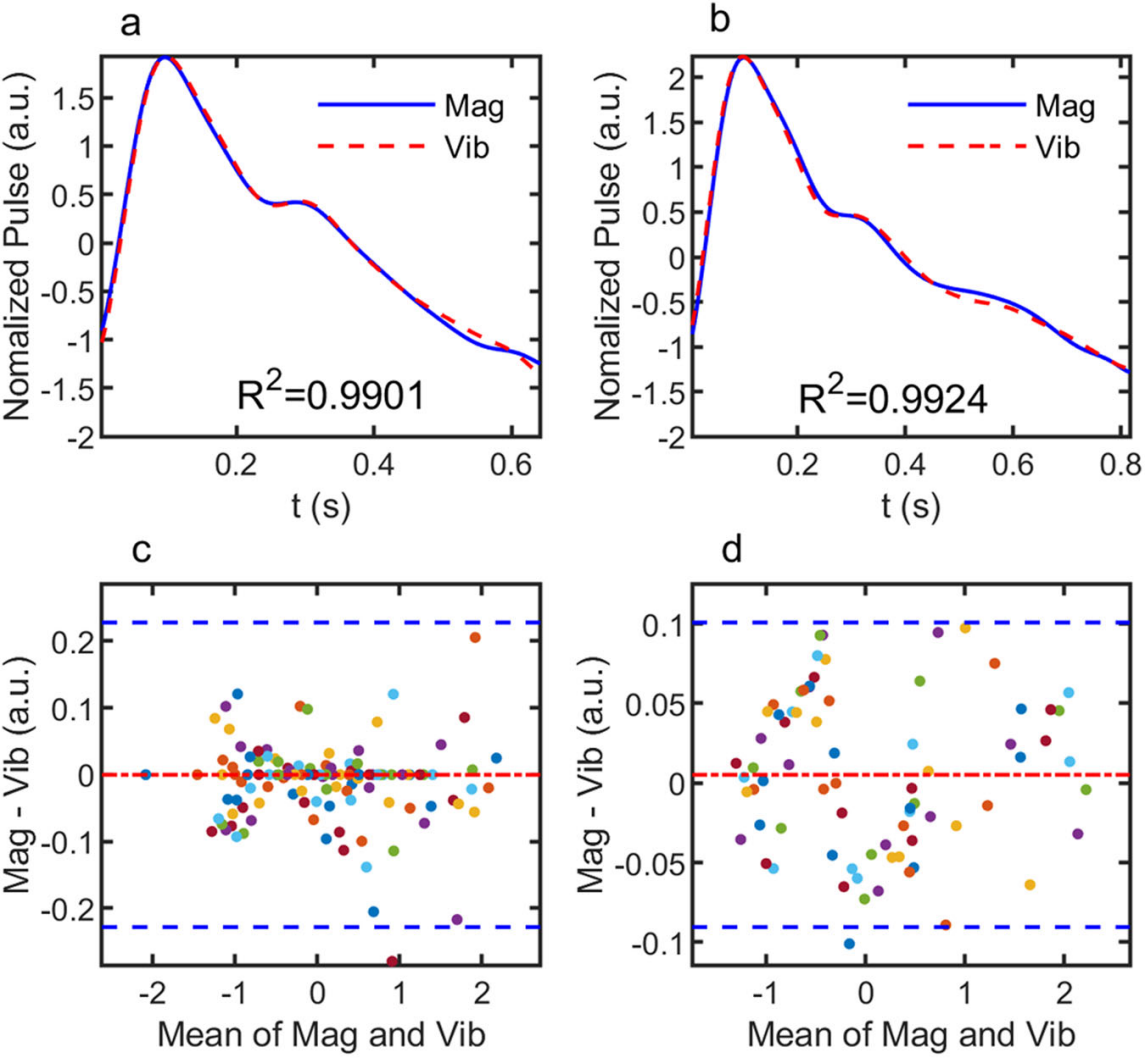
Correlation analysis of magnetic and vibrational signals. **a**, **b** Comparison of single pulse signal magnetic and vibrational signals. The vibrational signals are measured using VibroFlex QTec (**a**) and VibroGo (**b**), respectively. **c**, **d** Bland–Altman plots based on repeated measurements using VibroFlex QTec (**c**) and VibroGo (**d**). The two blue dashed-lines denote ±1.96 SD, and the red dash-dotted line denotes mean bias of difference between the two signals.

To have a more quantitative comparison, we compare normalized magnetic and vibrational data and perform Bland–Altman analysis in Fig. 4. The Bland-Altman plot is often used to analyze the matching between two methods of measurements for the same variable. It plots the difference against the mean of two sets of measurement values, highlighting the degree of agreement between the two measurement methods. The recommended limit of agreement is that 95% of the data points should lie within ±1.96 times of standard deviation (SD) of the mean difference^24,25^. Figure 4a, b show the average single pulse of magnetic (blue solid-line) and vibrational signal (red dotted-line) measured simultaneously from subject B. The vibrational signals are obtained using two different models of vibrometers: VibroFlex QTec for Fig. 4a and VibroGo for Fig. 4b. Other than a slight difference in surface roughness requirements, there are no key technical differences between the two models of vibrometers. The main purpose of using two different models of vibrometer is to check the correlation between magnetic and vibrational signals. The correlation coefficient between magnetic and vibrational signals in Fig. 4a is 0.99, indicating the same origin of the two signals. To quantify the difference statistically, we add more datasets to produce the Bland-Altman plot in Fig. 4c. The two dashed-lines denote ±1.96 SD. Except for a few outliers, the majority of the data points fall within the difference range of −0.15 to 0.12, with a mean bias of −$2.5\times {10}^{-4}$. Similar results are obtained for signals measured by the VibroGo, with a correlation coefficient of 0.99, and difference between −0.09 and 0.1 and mean bias of 5.1 × 10^−3^ in the Bland–Altman plot (Fig. 4d). The maximum deviation between the two measurement methods is 6.77% and 2.99%, respectively, for vibration measurements using VibroFlex QTec and VibroGo. In addition to the use of different types of vibrometer, we have also repeated the measurements using different types of magnetic sensors. These results suggest that the close correlation between magnetic and vibrational signal is a generic phenomenon which does not depend on the vibrometer or magnetic sensor used to measure the signals.

### Analytical model of MDVS sensor

In what follows, we examine more quantitatively the correlation between magnetic and vibrational signals using both analytical models and simulation. As discussed in the introduction, the MPG signal was initially interpreted as magnetic signature induced by blood flow. Later studies suggest that change of magnetic sensor’s detection axis caused by blood flow may also play a role, but quantitative understanding is lacking. In the present setup, the magnets are attached to a fingertip which consists of nail, skin, muscle, bones, arterial/vein vessels, and capillaries^26^. When the blood flows through the vessels, it generates pressure wave on the vessel walls^27^, which can propagate through the thick tissue and cause vibration of the skin. Therefore, without losing generality, the output signal of the magnetic sensor may be expressed as

1 $$\triangle {V}_{{{{{{\rm{mag}}}}}}}=S{{{{{\bf{s}}}}}}\cdot \left(\frac{\partial {{{{{\bf{H}}}}}}}{\partial \left({{{{{{\bf{r}}}}}}}_{{{{{{\rm{s}}}}}}}-{{{{{{\bf{r}}}}}}}_{{{{{{\rm{m}}}}}}}\right)}\triangle \left({{{{{{\bf{r}}}}}}}_{{{{{{\rm{s}}}}}}}-{{{{{{\bf{r}}}}}}}_{{{{{{\rm{m}}}}}}}\right)+\frac{\partial {{{{{\bf{H}}}}}}}{\partial {{{{{\bf{m}}}}}}}\triangle {{{{{\bf{m}}}}}}+\frac{\partial {{{{{\bf{H}}}}}}}{\partial \chi }\triangle \chi \right)$$

where $S$ is the sensitivity of the sensor (1.28 mV Oe^−1^ in the prototype device) and ${{{{{\bf{s}}}}}}$ is its sensing axis direction, ${{{{{\bf{H}}}}}}$ is the magnetic field at the sensor location, ${{{{{{\bf{r}}}}}}}_{{{{{{\rm{m}}}}}}}$ (${{{{{{\bf{r}}}}}}}_{{{{{{\rm{s}}}}}}}$) is the position of the magnet (sensor), ${{{{{\bf{m}}}}}}$ is the magnetic moment of the magnet (0.378 Am^2^ in the present case), $\chi$ is the magnetic susceptibility of the blood, and $\triangle ({{{{{{\bf{r}}}}}}}_{{{{{{\rm{s}}}}}}}-{{{{{{\bf{r}}}}}}}_{{{{{{\rm{m}}}}}}})$, $\triangle {{{{{\bf{m}}}}}}$, and $\triangle \chi$ are the corresponding changes induced by the blood flow. The first two terms of Eq. (1) correspond to signals induce by parallel displacement and rotation of the magnet, respectively, whereas the last term is due to change in magnetic susceptibility of the blood. Before performing numerical simulations to calculate the overall signal, it is instructive to examine the first two terms analytically using the magnetic dipole approximation, which is valid when the distance between the magnet and sensor $|{{{{{{\bf{r}}}}}}}_{{{{{{\rm{s}}}}}}}-{{{{{{\bf{r}}}}}}}_{{{{{{\rm{m}}}}}}}|$ (15 mm in the present setup) is much larger than the size of the magnet.

### Signal due to magnet displacement

We first consider the effect of parallel displacement of the magnet, i.e., $\triangle {{{{{\bf{m}}}}}}=0$. For simplicity, we assume that the sensor is stationary, and only one of the magnets is moving, which describes well the actual measurement setup. In this case, we may denote the magnet position as ${{{{{\bf{r}}}}}}=(x,{y},{z})$ in a Cartesian coordinate system with its origin at the sensor position. Then, the output signal may be written as $\triangle {V}_{{{{{{\rm{mag}}}}}}}^{{{{{{\rm{D}}}}}}}=S{{{{{\bf{s}}}}}}\cdot \frac{\partial {{{{{\bf{H}}}}}}}{\partial {{{{{\bf{r}}}}}}}\triangle {{{{{\bf{r}}}}}}$, where ${{{{{\bf{H}}}}}}=\frac{1}{4{{{{{\rm{\pi }}}}}}}(\frac{3{{{{{\bf{r}}}}}}({{{{{\bf{m}}}}}}\cdot {{{{{\bf{r}}}}}})}{{|{{{{{\bf{r}}}}}}|}^{5}}-\frac{{{{{{\bf{m}}}}}}}{{|{{{{{\bf{r}}}}}}|}^{3}})$ and $\triangle {{{{{\bf{r}}}}}}=(\triangle x,\,\triangle y,\,\triangle z)$. When the detection axis of the sensor and dipole moment of the magnet are aligned along *x*- and *z*-axis, respectively, i.e., ${{{{{\bf{s}}}}}}=({{{{\mathrm{1,0,0}}}}})$, ${{{{{\bf{m}}}}}}=({{{{\mathrm{0,0,1}}}}}){{{\rm{m}}}}$, $\triangle {V}_{{{{{{\rm{mag}}}}}}}^{{{{{{\rm{D}}}}}}}$ is calculated as

2 $$\triangle {V}_{{{{{{\rm{mag}}}}}}}^{{{{{{\rm{D}}}}}}}=\frac{3{mS}}{4{{{{{\rm{\pi }}}}}}}\frac{1}{{r}^{7}}[\left({x}^{3}+x{y}^{2}-4x{z}^{2}\right)\triangle z+\left({z}^{3}+z{y}^{2}-4z{x}^{2}\right)\triangle x-5{xyz}\triangle y],$$

where $r={({x}^{2}+{y}^{2}+{z}^{2})}^{1/2}$. When $x\gg z,y$, which represents the actual experimental setup, $\triangle {V}_{{{{{{\rm{mag}}}}}}}^{{{{{{\rm{D}}}}}}}$ is approximately given by $\triangle {V}_{{{{{{\rm{mag}}}}}}}^{{{{{{\rm{D}}}}}}}\approx (3{mS}/4{{{{{\rm{\pi }}}}}})(\Delta z/{x}^{4})$. On the other hand, when the laser beam is aligned in z-direction, $\triangle z$ is nothing else but the displacement measured by the vibrometer. Therefore, the first term of Eq. (1) is directly proportional to the vibration signal. The difference, if any, is mainly caused by the transverse movement of the magnet, i.e., $\triangle x$ and $\triangle y$, with respect to the laser beam direction, which can be minimized by optimizing the sensor position with respect to the magnet, i.e., to make $x\gg z,y$.

### Signal due to magnet rotation

Next, we consider the contribution due to rotation of the dipole which is initially aligned in *z*-direction, i.e., ${{{{{\bf{m}}}}}}=\left({{{{\mathrm{0,0,1}}}}}\right)m$. Without losing generality, we assume that the yaw, pitch and roll angles are $\alpha$, $\beta$, and $\gamma$, respectively, and the change of ${{{{{\bf{m}}}}}}$ due to rotation is given by $\triangle {{{{{\bf{m}}}}}}=R\left(\alpha ,\beta ,\gamma \right){{{{{\bf{m}}}}}}-{{{{{\bf{m}}}}}}$, where $R\left(\alpha ,\beta ,\gamma \right)$ is the rotation matrix. The signal induced by the dipole rotation can be calculated directly from Eq. (1), which reads

3 $$\triangle {V}_{{{{{{\rm{mag}}}}}}}^{{{{{{\rm{R}}}}}}}=\frac{3S}{4{{{{{\rm{\pi }}}}}}}\frac{1}{{r}^{5}}\left[\left({x}^{2}-\frac{{r}^{2}}{3}\right)\triangle {m}_{x}+{xy}\triangle {m}_{y}+{xz}\triangle {m}_{z}\right],$$

where $\triangle {m}_{x}$, $\triangle {m}_{y}$, and $\triangle {m}_{z}$ are the changes of magnetic moment in the three coordinate axis directions. When $\alpha ,\beta ,$ ${{{{{\rm{and}}}}}}$ $\gamma$ are very small and $x$ is much larger than $z$ and $y$, $\triangle {V}_{{{{{{\rm{mag}}}}}}}^{{{{{{\rm{R}}}}}}}$ is approximately given by $\triangle {V}_{{{{{{\rm{mag}}}}}}}^{{{{{{\rm{R}}}}}}}=({Sm}/2{{{{{\rm{\pi }}}}}})(\beta /{x}^{3})$. The result shows that the magnetic signal induced by the rotation is directly proportional to the pitch angle $\beta$, i.e., the angle of rotation around y-axis. Although the dipole approximation is valid in calculating the magnetic signal, to estimate the displacement signal measured by the vibrometer, we have to consider the finite size of the magnet. Here, we assume that the laser beam is initially focused at ${{{{{\bf{L}}}}}}=({L}_{x},\,{L}_{y},{L}_{z})$ on the top surface of the magnet with respect to the rotational center and its direction is parallel to *z*-axis. In this case, the displacement in *z*-direction is given by $\triangle {L}_{z}={{{{{\bf{k}}}}}}\cdot \left(R\left(\alpha ,\beta ,\gamma \right){{{{{\bf{L}}}}}}-{{{{{\bf{L}}}}}}\right)$ and when $\alpha ,\beta ,$ and $\gamma$ are small, it reduces to $\triangle {L}_{z}\approx \gamma {L}_{y}-\beta {L}_{x}$. Therefore, the displacement signal contains two components which are proportional to the pitch and roll angles, respectively. In other words, depending on the rotation directions, the displacement measured by the vibrometer may not be exactly the same as that of the output signal from the magnetic sensors. The ratio between $\triangle {V}_{{{{{{\rm{mag}}}}}}}^{{{{{{\rm{D}}}}}}}$ and $\triangle {V}_{{{{{{\rm{mag}}}}}}}^{{{{{{\rm{R}}}}}}}$ is on the order of $(\triangle z/x)/\beta$. Based on the displacement measured by the vibrometer, $\triangle z/x$ is on the order of ${10}^{-3}-{10}^{-2}$, which corresponds to a rotation angle of ${0.06} \deg -\,{0.6}{\deg}$, which means that $\triangle {V}_{{{{{{\rm{mag}}}}}}}^{{{{{{\rm{R}}}}}}}$ can be comparable to or even larger than $\triangle {V}_{{{{{{\rm{mag}}}}}}}^{{{{{{\rm{D}}}}}}}$, depending on the particular measurement setup. This may explain why the agreement between measured magnetic and vibration signals is generally better in the systolic than the diastolic half-cycle of the heartbeat (Figs. 2 and 4), as the sudden decrease of blood pressure after the systolic peak may result in not just parallel displacement but also rotation of the magnet.

### Numerical simulation of MDVS signal

**Fig. 5:**
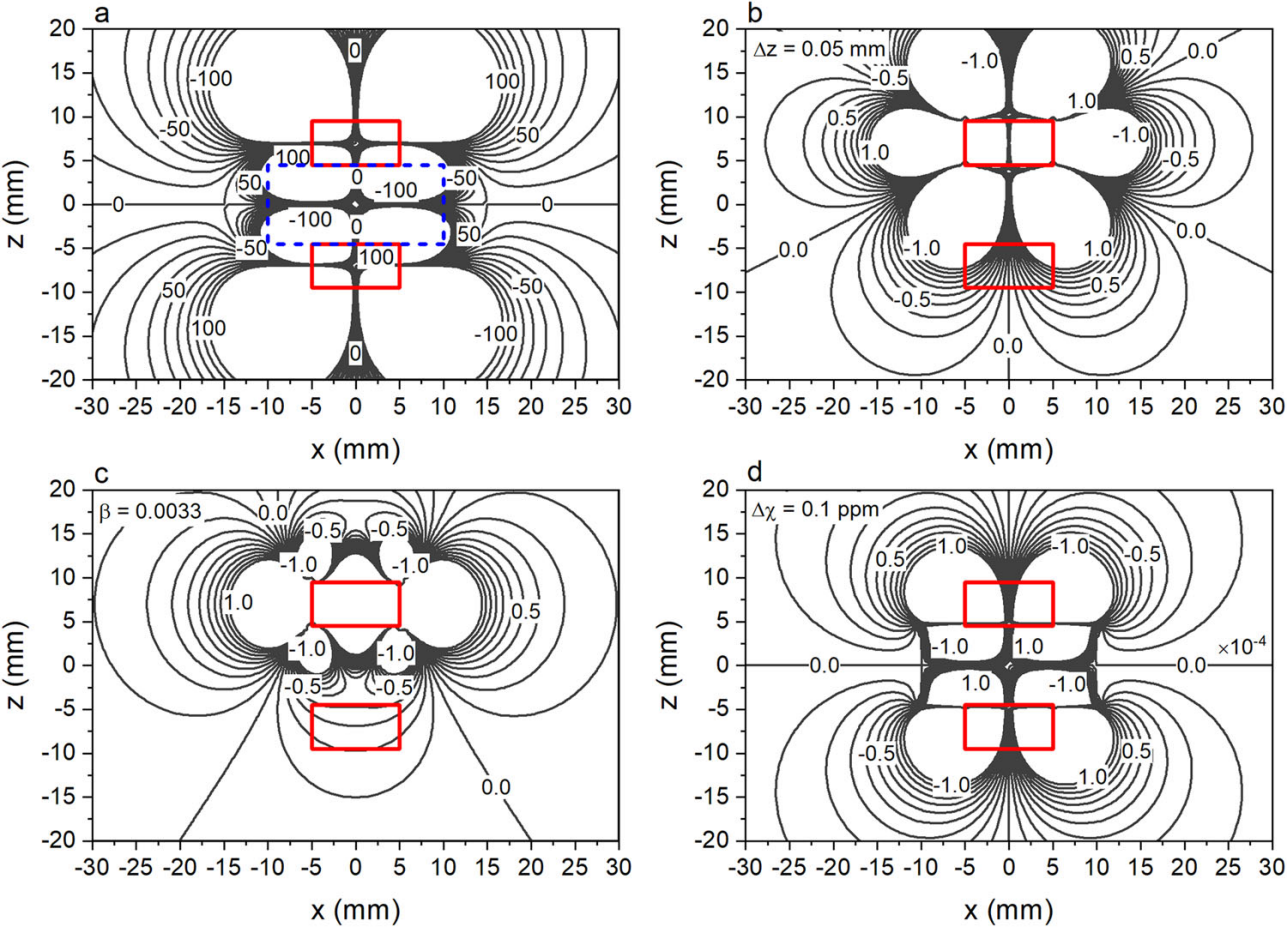
Simulated stationary field distribution and field change caused by magnet movement on the xz plane. Contour plots of **a** ${H}_{x}$ in the range of ±100 Oe, **b** ${\triangle H}_{x}$ caused by vertical shift of the top magnet by 50 μm, **c** ${\triangle H}_{x}$ caused by a pitch rotation of 0.0033 rad, and **d** ${\triangle H}_{x}$ induced by change of blood susceptibility. Note that the unit of (**a**–**c**) is Oe, and that of (**d**) is ${10}^{-4}$ Oe. Red boxes indicate the position of two cylindrical magnets and blue dashed box in (**a**) shows the size and position of finger cuboid.

Bearing in mind these analytical results, we now calculate the contributions of each term in Eq. (1) numerically under typical measurement conditions using the Software for Multiphysics Simulation (COMSOL). To facilitate the discussion, we re-set the coordinate system as follows (as we now have two magnets): *x*-axis and *y*-axis are parallel and transverse to the finger, respectively, *z*-axis is perpendicular to the cylindrical magnet surface, and the coordinate origin is at the middle of the magnet with *z*-axis passing through the rotational center of both magnets. Figure 5a shows the calculated contour lines of ${H}_{x}$ on the *xz* plane in the range of ±100 Oe, superimposed with the cross-sections of the magnets and fingertip. The two small rectangular boxes denote the cylindrical magnets with thickness $T$ = 5 mm, radius $R$ = 5 mm, and saturation magnetization ${M}_{{{{{{\rm{s}}}}}}}$ = 962.9 kA m^−1^. The large rectangular box defined by ${|x|}\le 10$ and ${|z|}\le 4.5$ mm represents the fingertip with ${|y|}\le 5$ mm. The spacing between the bottom surface of the upper magnet and top surface of lower magnet is initially set at 9 mm. The bottom magnet is fixed, whereas the top magnet is movable in response to the skin movement. The magnetizations of both magnets are pointing in the positive *z*-direction.

To maximize the detection signal, the sensor must be placed at a location where the field strength is below the sensor’s dynamics range (criterion 1) and at the same time the change caused by sensor displacement or rotation is largest (criterion 2). For the particular senor used in the experiment which has a dynamic range of ±100 Oe, the simulation results in Fig. 5a show that criterion 1 is satisfied as long as the sensor is placed in the region where the contour lines are shown ($|{H}_{x}| < 100\,{{{{{\rm{Oe}}}}}}$). However, when the sensor is too far away from the magnet, it may not be able to detect the small change of field caused by the blood flow, which may originate from magnetic displacement/rotation or blood susceptibility change, or the combination of all these factors. We first calculate the field change ($\triangle {H}_{x}$) caused by magnet displacement or rotation under typical measurement conditions. Figure 5b shows the contour of $\triangle {H}_{x}$ caused by the movement of the top magnet in *z*-direction by 50 μm, which corresponds to the typical displacement of magnet measured by the vibrometer due to blood flow. Similarly, Fig. 5c shows the change caused by rotating the magnetization of the top magnet by $3.3\times {10}^{-3}$ rad away from *z*-axis around *y*-axis (i.e., $\beta ={0.19}{\deg}$). The rotation angle is chosen such that the maximum deflection of the edge of the magnet is comparable to the vertical displacement used for obtaining the results in Fig. 5b. From both figures, we can see that maximum change appears along the middle line of the top magnet in *x*-direction with its magnitude decreasing quickly away from the magnet. Within the range of $15\,{{{{{\rm{mm}}}}}} < \left|x\right| < 20\,{{{{{\rm{mm}}}}}}$ and $5\,{{{{{\rm{mm}}}}}} < z < 10\,{{{{{\rm{mm}}}}}}$, it is possible to obtain a change of 0.5–1 Oe for ${H}_{x}$, which is detectable by the sensor.

We next simulate the field change caused by susceptibility change of the blood. Due to the difference in magnetic responses of HbO_2_ and Hb, the magnetic susceptibility of oxygenated and de-oxygenated blood varies slightly,^7^ and its difference relative to water $\Delta \chi$ can be approximated as follows^28^:

4 $$\Delta \chi ={{{{{\rm{Hct}}}}}}\left(\Delta {\chi }_{{{{{{\rm{do}}}}}}}\left(1-{{{{{\rm{Hb}}}}}}{{{{{{\rm{O}}}}}}}_{2}\right)+\Delta {\chi }_{{{{{{\rm{oxy}}}}}}}\right),$$

where ${{{{{\rm{Hct}}}}}}$ is the volume fraction of red blood cell, $\Delta {\chi }_{{{{{{\rm{oxy}}}}}}}$ is the susceptibility difference between fully oxygenated blood and water, $\Delta {\chi }_{{{{{{\rm{do}}}}}}}$ is the susceptibility difference between fully oxygenated and deoxygenated red blood cells, and HbO_2_ ranges from $0$ (fully deoxygenated) to $1$ (fully oxygenated)^28–31^ According to Jain et al., $\Delta \chi /{{{{{\rm{Hct}}}}}}$ is about $-2\times {10}^{-8}$ at fully oxygenated sate and $2.5\times {10}^{-7}$ at deoxygenated stated. Since the magnetic susceptibility of water is $7.18\times {10}^{-7}$ and ${{{{{\rm{Hct}}}}}}$ is around 0.4–0.5, the magnetic susceptibility of blood is between 2$.8\times {10}^{-7}-4.8\times {10}^{-7}$, depending on its oxygenation state. The maximum change is less than 0.1 ppm. To simulate its effect on field distribution, we calculate the field difference caused by the variation of magnetic susceptibility in the fingertip region by 0.1 ppm, and results are shown in Fig. 5d. As can be seen, the change in ${H}_{x}$ is on the order of ${10}^{-5}$Oe in the region of interest, i.e., $15\,{{{{{\rm{mm}}}}}} < \left|x\right| < 20\,{{{{{\rm{mm}}}}}}$ and $5\,{{{{{\rm{mm}}}}}} < z < 10\,{{{{{\rm{mm}}}}}}$, which is too small to be detected by the magnetic sensor used in this work (note: the unit in Fig. 5d is 10^−4 ^Oe). The above results demonstrate clearly that the pulsatile signals detected by the magnetic sensor are dominantly of mechanical origin, the MMSB contribution, if any, is negligible. It is worth pointing out that the calculated values can be considered as the upper bound of the blood contribution to detected magnetic signals as we assume that the fingertip is filled entirely by blood.

**Fig. 6:**
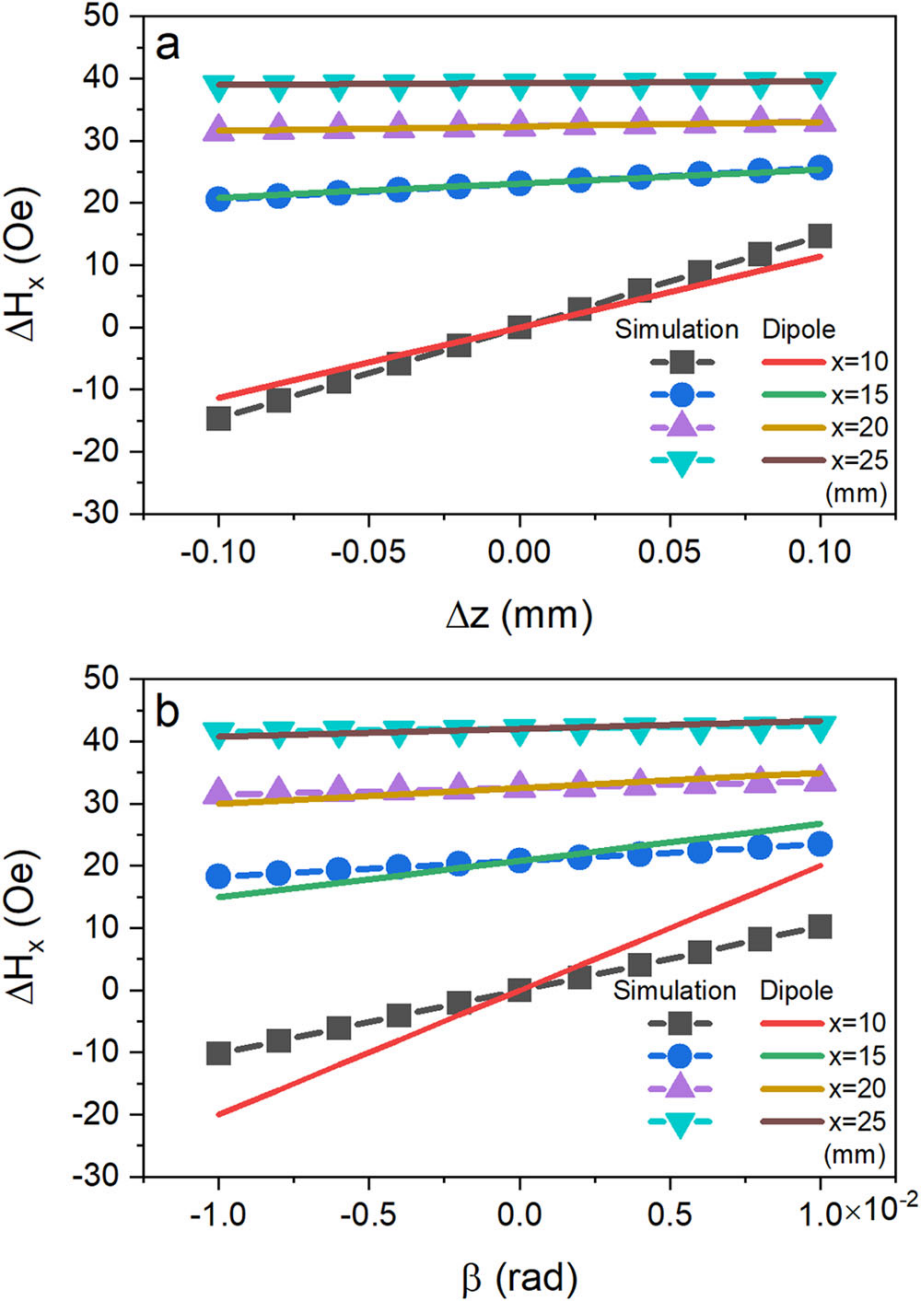
Comparison of field change obtained by COMSOL simulation (symbol) and dipole approximation (solid-line). **a** ${\triangle H}_{x}$ as a function of vertical shift $\triangle z$ and **b** ${\triangle H}_{x}$ as a function of pitch angle $\beta$ of the top magnet at different radial position, $x=$ 10, 15, 20, 25 mm, from the center of the magnet.

Before we end this section, we discuss the validity of the dipole approximation by comparing the analytical and COMSOL simulation results. Figure 6a shows $\triangle {H}_{x}$ obtained by the two methods as a function of vertical displacement of the top magnet ($\triangle z$) at different radial position ($x$) along the middle line of the magnet. Similar results are shown in Fig. 6b for pitch angle $\beta$. Solid-lines are results obtained from the dipole approximation and symbols are from COMSOL simulations. As expected, good agreements are obtained when both $\triangle z$ and $\beta$ are small, and $x$ is about 3 times larger than that of the radius of the magnet. Therefore, the dipole approximation can be used to optimize the position of the sensor with respect to the magnet.

## Conclusions

We have proposed and designed a pulse detection device which allows us to conduct a comparative study to determine the origin of so-called MPG signals through simultaneous measurements using a magnetic sensor and a vibrometer. Based on both experimental and simulation results, we conclude unambiguously that the detected signal is not originated from change of blood magnetic properties but rather from mechanical motion of the magnet induced by blood flow, including both vertical displacement and rotation. Apart from the determination of signal origin, the proposed fingertip-type magnetically detected vibration sensor is much more robust and simpler than the previously report wrist-type MPG sensor, and thus is more suitable for practical applications. It has clear advantages over existing pulse detectors in coping with unusual monitoring conditions. In addition to heart rate detection, its direct relevance with the blood pressure wave makes it promising for blood pressure measurement.

## Methods

### Signal processing and statistical analysis

The detected signals were processed with MATLAB as follows. First, the raw data with a sampling rate of 240 Hz was segmented into sequences with a duration of 30 s. Second, the raw data was filtered using a bandpass filter with a passband of 0.8–10 Hz to remove high-frequency noise and baseline shift. The bandpass filter was designed with a steepness of 0.85 and a stopband attenuation of 60 dB. Third, z-score analysis was performed to transform the two types of signals with different amplitudes and units into similar ranges. This converts the data to have a mean of zero and a standard deviation of one, allowing for direct comparison of the signals. The transformed signals are shown in Fig. 2a, d, g. Fourth, single pulses were extracted by averaging all pulses within the period of the data sequence. The resulting signals are shown in Fig. 2b, e, h. To further analyze the characteristics of the single pulse signals, the second derivative of the signals was computed and is shown in Fig. 2c, f. Finally, to assess the agreement between the single pulse signals obtained from different sequences, Bland–Altman plot analysis was performed based on 60 pulses from three sequences of signals. This method is commonly used to assess the agreement between two measurement techniques and is based on the difference between the two measurements plotted against their mean. The results of the Bland–Altman plot analysis provide insight into the consistency and variability of the single pulse signals obtained from different sequences.

### Simulation setup

Simulation of magnetic signals was performed using COMSOL Multiphysics® software. The simulation was performed with an initial distance of 14 mm between the body centers of the two magnets, and a finger diameter of 9 mm was used. To simulate the effect of vertical vibration on the finger, the position of the magnet in the positive *z*-axis was varied to model the expansion of the finger due to blood circulation. As an approximation, a cuboid was used to simulate the susceptibility change inside the finger. The dimensions of the cuboid were set to be 20 mm along the *x*-axis, 10 mm along the *y*-axis, and 9 mm in height. This allowed for the accurate representation of the magnetic fields within the finger and the effects of different movements on the magnetic signals. When simulating the effect of tilt of magnet, only the magnet in the positive *z*-axis was rotated around the center of the bottom surface, which accurately represents the actual situation during measurements.

### Supplementary information

Supplementary Information

## Data Availability

The data that support the findings of this study are available from the corresponding author upon reasonable request.
