# Supplementary material for: A fingertip-type magnetic pulse detection device for unusual monitoring conditions
Source: Commun Eng. 2023 Jul 28;2:52. doi: 10.1038/s44172-023-00102-2 (PMC10955891; doi:10.1038/s44172-023-00102-2)
Supplement: Supplementary file 1 — Supplementary Information [file 44172_2023_102_MOESM1_ESM.pdf]

Supplementary Note 1

As we discussed in the main text, different types of magnetic sensors can be used to detect the pulse signal provided they have suitable dynamic range and sensitivity. The actual dynamic range requirement is determined by the magnets used and the spacing between the sensor and magnets. To facilitate the fabrication of compact prototype devices, the results described in the main text were acquired using commercial devices with proper packaging. As shown Fig. S1 below, clear signals were also obtained from home-made sensors without packaging. The pulse waveform shown in Fig.1S(a) was obtained from spin Hall magnetoresistance (SMR) sensor[1], whereas the one in Fig.S1(b) was acquired using an anomalous Hall sensor with a superparamagnetic sensing layer[2].

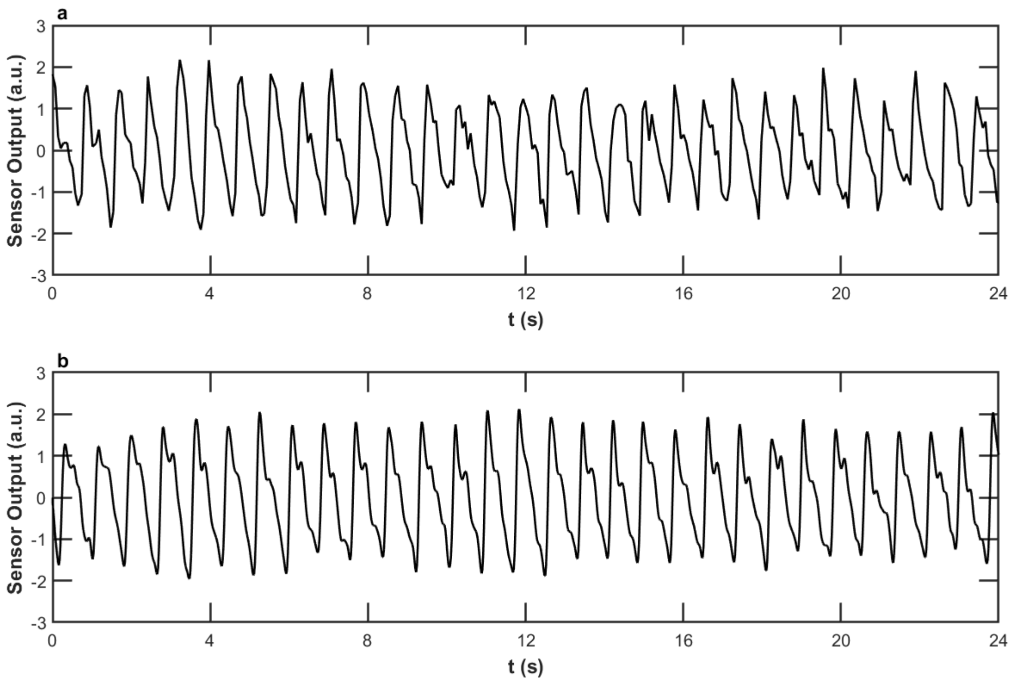

Supplementary Figure 1. Pulse waveform collected using (a) spin Hall magnetoresistance sensor and (b) anomalous Hall effect sensor from the same subject whose pulse data were presented in Fig.2 d-i of the main text.

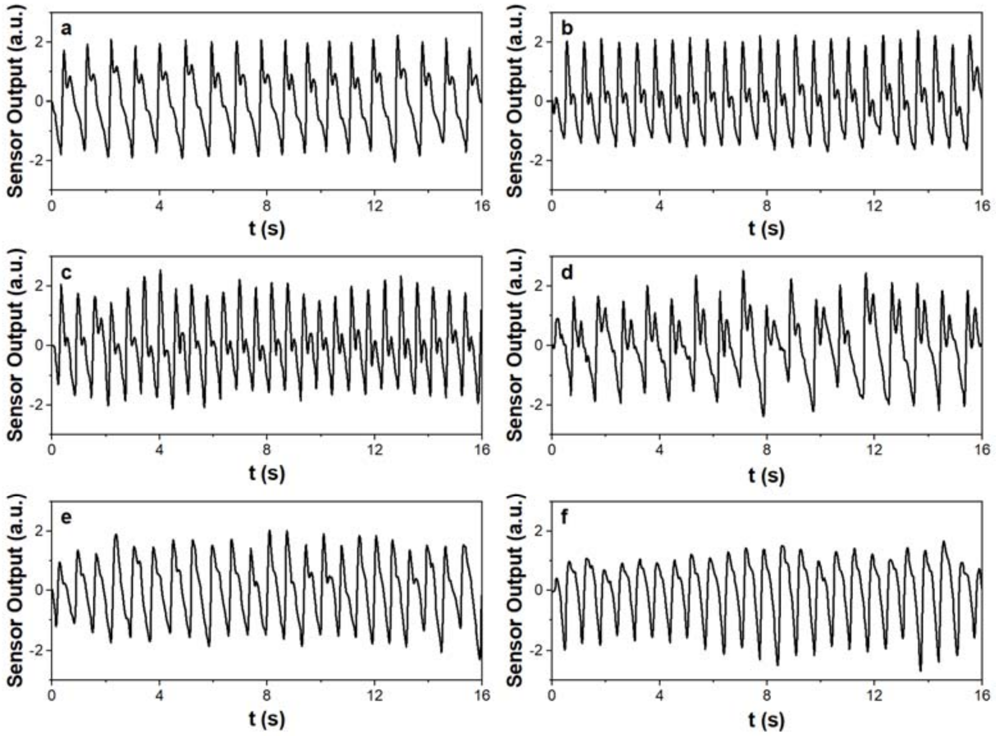

Supplementary Figure 2. Pulse waveform collected from different subjects: (a), (b) male in their 20s; (c), (d) male in their 30s; (e) male in 40s; (f) female in 20s.

Supplementary References:

1 Xu, Y., Yang, Y., Zhang, M., Luo, Z. & Wu, Y. Ultrathin All-in-One Spin Hall Magnetic Sensor with Built-In AC Excitation Enabled by Spin Current. *Advanced Materials Technologies* **3**, 1800073 (2018).

2 Chen, X., Xie, H., Shen, H. & Wu, Y. Vector Magnetometer Based on a Single Spin-Orbit-Torque Anomalous-Hall Device. *Physical Review Applied* **18**, 024010 (2022).
